# Solving Problems in Social–Ecological Systems: Definition, Practice and Barriers of Transdisciplinary Research

**DOI:** 10.1007/s13280-012-0372-4

**Published:** 2013-03-10

**Authors:** Per Angelstam, Kjell Andersson, Matilda Annerstedt, Robert Axelsson, Marine Elbakidze, Pablo Garrido, Patrik Grahn, K. Ingemar Jönsson, Simen Pedersen, Peter Schlyter, Erik Skärbäck, Mike Smith, Ingrid Stjernquist

**Affiliations:** 1 Faculty of Forest Sciences, School for Forest Management, Swedish University of Agricultural Sciences, PO Box 43, 730 91 Skinnskatteberg, Sweden; 2 Department of Work Science, Business Economics & Environmental Psychology, Faculty of Landscape Planning, Horticulture and Agricultural Sciences, Swedish University of Agricultural Sciences, Box 88, 230 53 Alnarp, Sweden; 3 Faculty of Forest Sciences, School for Forest Management, Swedish University of Agricultural Sciences, PO Box 43, 739 21 Skinnskatteberg, Sweden; 4 School of Education and Environment, Kristianstad University, 291 88 Kristianstad, Sweden; 5 Department of Forestry and Wildlife Management, Faculty of Applied Ecology and Agricultural Sciences, Hedmark University College, 2480 Evenstad, Norway; 6 Environmental and Resource, Dynamics Group, Department of Physical Geography and Quaternary Geology, Stockholm University, 106 91 Stockholm, Sweden; 7 Forest Research, Northern Research Station, Centre for Human and Ecological Sciences, Roslin, Midlothian, EH25 9SY UK

**Keywords:** Research policy, Sustainable development, Sustainability, Knowledge production, Learning, Green infrastructure

## Abstract

Translating policies about sustainable development as a social process and sustainability outcomes into the real world of social–ecological systems involves several challenges. Hence, research policies advocate improved innovative problem-solving capacity. One approach is transdisciplinary research that integrates research disciplines, as well as researchers and practitioners. Drawing upon 14 experiences of problem-solving, we used group modeling to map perceived barriers and bridges for researchers’ and practitioners’ joint knowledge production and learning towards transdisciplinary research. The analysis indicated that the transdisciplinary research process is influenced by (1) the amount of traditional disciplinary formal and informal control, (2) adaptation of project applications to fill the transdisciplinary research agenda, (3) stakeholder participation, and (4) functional team building/development based on self-reflection and experienced leadership. Focusing on implementation of green infrastructure policy as a common denominator for the delivery of ecosystem services and human well-being, we discuss how to diagnose social–ecological systems, and use knowledge production and collaborative learning as treatments.

**Electronic supplementary material:**

The online version of this article (doi:10.1007/s13280-012-0372-4) contains supplementary material, which is available to authorized users.

## Introduction

Historically, research policy has swung between a strong sector-focus on solving pre-defined problems and basic research with full academic freedom. Partly as a response to increased awareness of the complexity induced by interactions between human and natural systems at multiple scales, research policy in the European Union (EU) has evolved into research and innovation policies, where utilization of knowledge, implementation, and commercialization are emphasized (Anon. 2006). Increased competition through scientific quality, and innovation-based economic growth are two tools for implementation (e.g., Regeringens Proposition 2008/09, 2012/13). This is to be established by trans-national cooperation, frontier research, stimulation to enter into the profession of researcher, and by bringing science and society closer together (Anon. 2006, 2011).

Research and innovation are thus at the top of the EU’s agenda for growth and jobs, and Member States have been encouraged to yearly invest 3 % of their Gross Domestic Product in research and development by 2020. The central role of research was recognized by the Lisbon European Council of 2000 (Anon. 2006), which established for the EU a new strategic goal for the next decade to become the most competitive and dynamic knowledge-based economy in the world, capable of sustainable economic growth with more and better jobs and greater social cohesion. Driven by the challenge to stabilize the financial and economic system in the short term, while also taking measures to create the economic opportunities of tomorrow, the EU’s new program Horizon 2020 for funding of research and innovation for 2014–2020 has been launched (European Commission 2011). It advocates that research and innovation shall help deliver jobs, prosperity, quality of life, and global public goods (see also Anon. 2011; Regeringens Proposition 2012/13). All sectors of the European economy are expected to benefit, including agriculture, fisheries and food, health, transport, energy—especially renewables—and information and communication technologies.

However, economic growth and increased competition have historically often resulted in negative impact on the environment (Marsh 1864; MEA 2005; Kumar 2010). Still, economic development, up to a point, is commonly correlated to higher levels of social sustainability (Birdsall 1993). Societal choice as to what and how much land and water should provide in terms of ecosystem services is increasingly complex, changing over time, and more unpredictable relative to the dynamics of natural processes (Sandström et al. 2011). Biophysical disturbances and their unclear effects linked to climate change are additional examples of uncertainty. To cope with all of these factors, the concepts of adaptive management (Lee 1993) and adaptive governance (Folke et al. 2005) have emerged. Realizing them requires explicit focus on integrated social and ecological systems when analyzing different aspects of ecosystem services (MEA 2005), as well as governance, institutions, and policy instruments (Norgaard 2010). The precautionary principle has also been discussed in terms of research and policies on interactions between environment and health (Harremoes et al. 2001; Martuzzi 2007).

The societal process of sustainable development (SD) towards sustainability on the ground as defined in policies, requires place and area-based solutions that integrate social and ecological systems in spaces and places (Grodzynskyi 2005; Angelstam et al. 2013a). SD is about stakeholders navigating together (Baker 2006) in all dimensions of sustainability. Given current risks and uncertainties, this requires adaptive governance that embraces the inherent complexity of landscapes as social–ecological systems. Adaptive governance can be understood as an institutional response to the challenges of the SD process towards sustainability. A key characteristic of adaptive governance is iterative learning that enables humans to cope with uncertainty and change, thus enabling institutions that guide stakeholder collaboration (Folke et al. 2005). This is in line with the social learning concept (Leeuwis and Pyburn 2002; Keen et al. 2005; Axelsson et al. 2013b), as well as with the concepts of inference towards the best explanation or best understanding (Harman 1965; Lipton 2004; Annerstedt 2010). Similarly, sustainability indicators and measurable variables need to be developed in collaboration with stakeholders and decision makers, and parameter values need to be defined as norms or performance targets. One example is evidence-based thresholds for organisms in relation to habitat loss (Angelstam et al. 2013c). If this is successful, both individual stakeholders and communities can assess their systems’ sustainability status and thus improve their ability to steer development toward an agreed desired state. This applies in principle to any criterion such as ecological (Villard and Jonsson 2009), economic (Barnes 2006), social, and cultural (Axelsson et al. 2013a). In a similar fashion, stakeholder-based modeling can allow identification of conflicts and movement towards the development of joint improved systems for common understanding. This alleviates identification of strategies to further local resource governance and management by identifying knowledge needs of local communities (Sverdrup et al. 2010).

Ultimately, natural capital is a foundation for human well-being and quality of life (Neumayer 2010). To communicate the need for improved biodiversity conservation by promotion of ecosystem health and resilience for the provision of ecosystem services, the concept green infrastructure has emerged at EU and national policy levels (Naumann et al. 2011). Green infrastructure is a broad and multifunctional concept including both natural and semi-natural terrestrial and aquatic areas. Functional green infrastructures are crucial for the health, adaptive capacity, and resilience of ecosystems by providing space and structures to maintain or restore all their functions and to support adaptation to climate change effects (European Commission 2010). However, the policy vision of functional green infrastructures is in stark contrast to the current poor quality of habitat networks for human beings and other species. A key barrier is limited collaboration among actors and societal sectors (Angelstam et al. 2003; Blicharska et al. 2011). The same goes for research, where it is often argued that transdisciplinary studies would be the most adequate for approaching this type of complex phenomenon. The actual practice of such research is still relatively scarce, however, due both to limited funding and research organizations’ capacity.

To develop functional green infrastructures as an outcome of adaptive governance and management in landscapes it is thus urgently needed to (1) increase collaboration among academic and non-academic actors to facilitate learning and sharing of knowledge and experience (Sverdrup et al. 2010), and (2) develop methods for achieving evidence-based knowledge (Angelstam et al. 2004; Rockström et al. 2009), and (3) apply appropriate management (see Elbakidze et al. 2013). Additionally, approaches for spatial green infrastructure planning at scales from local to trans-national are needed to support the work of planners, managers, and other decision-makers that influence the landscape (Skärbäck 2007a, b; Andersson et al. 2012a).

Production of new knowledge and collaborative learning processes are two important dimensions of transdisciplinary research (Tress et al. 2006). The overall aim of this study is an attempt to define barriers and bridges for the transition from disciplinary academic research towards transdisciplinary research. What are the impediments to the development of a transdisciplinary research agenda? What factors influence functional transdisciplinary research team development? First, we summarize the differences between basic, applied, and transdisciplinary research. Second, based on 14 experiences from problem-solving real-world challenges, we used group modeling to map the perceived barriers and bridges for researchers to become involved with and be successful in transdisciplinary research. Finally, we discuss the importance of transdisciplinary research on green infrastructures for ecological sustainability and human well-being. We also elaborate on how the diversity of landscape concepts can be used as a tool to diagnose social–ecological systems and treatments by collaborative learning concerning functional green infrastructure development.

## Defining Transdisciplinary Research

Transdisciplinary research is one avenue among others to identify and learn about the SD process, and factors that influence sustainability. This form of research is based on integration of multiple disciplines and the active inclusion and participation of stakeholders representing different societal sectors in the processes of problem formulation, knowledge production, and learning (Tress et al. 2006; Hirsch Hadorn et al. 2008; Klein 2008; Axelsson 2010; Axelsson et al. 2011). To succeed with this, global (i.e., biophysical), social, and human systems need to be considered simultaneously (sensu Komiyama et al. 2011). Including the social system means understanding the needs and interests of different stakeholders, but also to understand the interconnectedness with the regional, national, and international levels of societal steering. Finally, the human system includes life style, health as well as values and norms among people. The diversity of landscape concepts is useful as a tool for integration of different research disciplines and actors in the triangle of knowledge—education, research, and innovation—with the aim to develop new approaches to SD and sustainability (Grodzynskyi 2005; Angelstam et al. 2013b).

Media and other expressions of society’s views are crucial to understand ecological and social processes, natural resource management, governance and consequential effects on health and behavior input from public debate. This information should also be incorporated into the research process. Hence researchers and stakeholders will bring in their expertise in a collaborative learning process (Daniels and Walker 2001), and develop a framework to produce the required new knowledge. Some partners in the process will contribute with their disciplinary expertise, whereas others will take inter- or transdisciplinary perspectives. The concept of knowledge production thus includes both the production of new knowledge and learning processes (Gibbons et al. 1994).

Suggested research techniques stress the need for cooperative investigations in order to detangle for example mechanisms behind diseases related to ecological change (Plowright et al. 2008). These techniques and other recent scientific attempts to approach questions of complexity in social–ecological systems demonstrate the irrelevance of talking only in terms of basic and applied science. In basic research the main motivational force is usually considered to be the researcher’s curiosity and wish to expand the knowledge related to a certain topic. This has traditionally been in opposition to applied research, where the motivation is to solve practical problems of the modern world rather than to actually expand knowledge as such. In a transdisciplinary research process the joint problem formulation (dealing with observations, theories, and experiences in a non-hierarchical manner) is fundamental, as well as the inference technique and the iterations of the process (Hirsch Hadorn et al. 2008). This distances the concept from being either basic or applied (Table 1).

**Table 1:** Overview of characteristics of basic, applied, and transdisciplinary research (after Hirsch Hadorn et al. 2008)

Type of research	Disciplines	Problem	Stakeholder
Basic	One discipline	Defined by researcher	Not involved
Applied	One or more discipline	Defined by stakeholder/s	One or several
Transdisciplinary	Several disciplines as defined by the problem	Defined jointly by researchers and stakeholders	Several as defined by the problem

Transdisciplinary research thus needs to be considered as an applied practice, evolving from current problems of the world that needs to be practically solved, and not attached to pre-established method or design. Rather, these will evolve throughout the continuous work and collaboration between researchers from different scientific disciplines, stakeholder participation, as well as communication and dissemination (e.g., Angelstam et al. 2013a). Eventually, if successful, the process will result in joint problem–solution, across sciences, technology, and society (Galliers 2004; Annerstedt 2010). This results in a team approach to problem-solving research that aims for synergy from the phases of problem definition to solutions. Consequently, this will enhance integration of novel theoretical and innovative methodological perspectives from different disciplines, as well as including non-academic knowledge in the empirical problem-solving process (Leavy 2011).

## Barriers and Bridges to Transdisciplinary Research

### Drawing upon Multiple Case Studies by Group Modeling

What are the impediments to the development of a transdisciplinary research agenda? We used systems thinking and a generic group modeling procedure (Vennix 1996; Maani and Cavana 2000; Sterman 2000; Nguyen et al. 2011) to model the authors’ experiences of attempts to solve complex real-world problems (see Hirsch Hadorn et al. 2008) to answer this question. Our author collective includes members that range from those just embarking on the process of transdisciplinary research to those with long experience. The dynamic and iterative character of transdisciplinary research provides opportunities for mutual learning, joint activities, and feedback relationships. Something that may eventually result in a mutual language of concepts and models that could be used in specialized and societal contexts. Previous research in ecology has presented the idea of inferring conclusions from unique case studies (Shrader-Frechette and Earl 1994). In this study we present our pooled experiences from 14 case studies representing complex real-world problems.

**Fig. 1:**
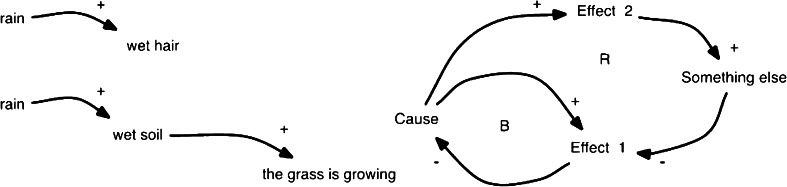
Simple cause–effect relationships shown as causal loop diagrams (CLD). The variable at the tail of the arrow causes a change to the variable at the head. A *plus sign* indicates that the variable at the tail and the variable at the head of the arrow change in the same direction, while a *minus sign* indicates that the variables at the tail and head change in opposite directions. Thus, if the variable at the tail increases, the variable at the head decreases and vice versa. The letter *R* in the middle of a loop indicates that the loop is reinforcing, causing either a systematic growth or decline. The letter *B* indicates that the loop is balancing and moves the system towards equilibrium. Thus, (i) The rain irrigates the soil, which is needed for the grass to grow. Another effect of the rain is that my hair becomes wet. The growth of the grass and the wet hair seem to be correlated due to the same cause but the grass does not grow because my hair is wet. Even if the phenomena are statistically correlated, the cause–effect relationship is not sound. (ii) A cause–effect relationship with two counteracting factors acting on effect 1

Causal loop diagramming (CLD) methodology was used to map and analyze major system connections, important feedbacks and system structures affecting researchers’ and practitioners’ ability to become involved with and be successful in transdisciplinary research. A major advantage of the CLD notation is that it uses a common unambiguous language for describing relationships between components within a system, thus clearly communicating the construction of the system thereby facilitating peer review and quality control of the proposed system. The model development process is collaborative and dialectic, that is characterized by successive cycles of suggestions for important systems relationships, critical assessment and critique within the larger group and subsequent redevelopment and improvement. The outcome is a jointly developed, tested and accepted model, which is based on agreement of causal effects between components. This process necessitates all participants to be actively involved, carefully argumentative, and good listeners to others’ arguments and counter arguments. As a language, the CLD method is easily learned and it requires no advanced mathematical knowledge or specialized educational background (Hjorth and Bagheri 2006). The principle of causality is shown in Fig. 1.

### Experiences of Research Aimed at Solving Real-World Problems

The experiences that the group modeling was based on consisted of the authors’ experiences from working with different combinations of global (biophysical), social, and human systems (sensu Komiyama et al. 2011) (Table 2). First, an example illustrating primarily a global system involves top predators, herbivores, and their biophysical landscape. Interactions among these elements, and forest and wildlife managers, affect lichen and bird species that depend on deciduous trees species such old aspen and willow trees which are the preferred food of moose. Similarly, the re-colonization of wolves has negative effects on hunting as recreation, and the opportunity to keep grazing cattle and sheep to maintain the cultural landscape (Angelstam 2002). Second, an example of a social system problem is about how to mitigate negative effects of urbanization on the balance between human’s biological conditions by spatial planning of urban areas. This requires integration of stakeholders from different sectors at multiple levels, the development of visions and scenarios, which are expressed as maps (Andersson et al. 2012a, b). Finally, given changing profiles of human disease (Wittchen et al. 2011), a human system example is the need to focus on environmental psychology in terms of studies of how ecosystems affect human psychology and behavior. In biological terms, human behavior is determined by certain brain structures that are under the continuous influence of intra-organic feedback systems involving hormones and other transmitter substances, as well as of extra-organic input and stimuli.

**Table 2:** Overview of authors’ experiences of research aimed at solving real-world problems, and their global (i.e., biophysical), social, and human systems (see Komiyama et al. 2011). These case studies were used as a base for the CLD diagramming. For details, see Electronic Supplementary Material

Case study	
1	Trophic interactions among predators, prey and vegetation
2	Brown bears and forest reindeer herding in Lapland
3	Moose hunting and wolves in Norway
4	Protected area network functionality in Sweden
5	Spatial planning for habitat networks in Scotland
6	Swedish Environmental Objective “Magnificent Mountains”
7	Cultural and natural values in road planning
8	Geographic Information Systems and spatial planning
9	Land consolidation in Dalarna County, Sweden
10	Creation of the Roztochya Biosphere Reserve in Ukraine
11	Public procurement of food with an environmental profile
12	Landscape character vs. health and wellbeing
13	Stress, neurobiology, and green space management
14	Establishment of a rehabilitation garden

### Group Modeling Based on Case Studies of Problem-Solving

**Fig. 2:**
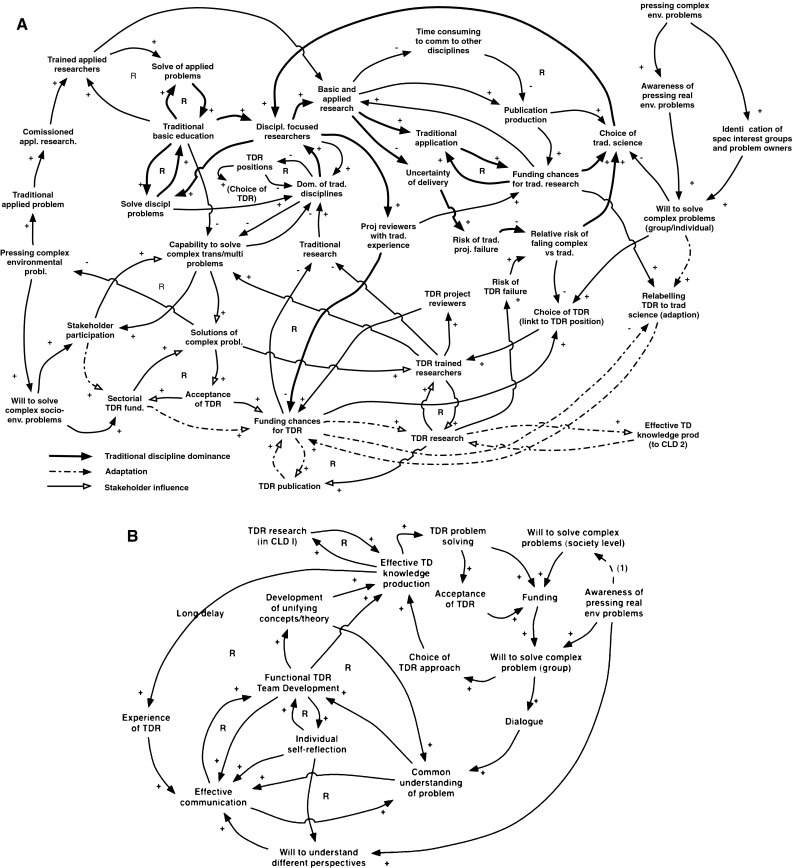
**a** Causal loop diagram (CLD1) that resulted from group modeling of the question “What are the impediments to the development of a transdisciplinary research agenda?, based on 14 case studies of problem-solving. **b** Causal loop diagram (CLD2) resulted from group modeling of the question “What factors influence functional TDR team development?”, based on 14 case studies of problem-solving

Group modeling based on the authors’ experiences of being involved with research aimed at solving real-world problems identified four key factors affecting the success and development of transdisciplinary research (Fig. 2a, b). These were (1) the degree of traditional disciplinary formal and informal control and dominance; (2) the degree to which researchers can frame project applications within a transdisciplinary research agenda while still remaining acceptable within a mainstream disciplinary peer review system; (3) the central role of stakeholder participation in all steps of the process, and (4) the importance of functional transdisciplinary research team development, which requires self-reflection as well as experienced leadership.

First, the current higher academic educational system reinforces traditional disciplinary approaches. The basic education therefore produces disciplinary trained researchers, who largely tend to focus on successfully solving disciplinary or applied pre-defined problems. As a consequence this subsystem is self-reinforcing. This bias affects funding chances for transdisciplinary research negatively as well as career choices, both of which reinforces the bias against transdisciplinary knowledge production.

Second, researchers interested in solving complex problems may choose to adapt to the disciplinary bias by producing applications that are, superficially, re-labeled to appear sufficiently traditional in approach to increase the chances of funding, thus allowing a transdisciplinary research agenda. From traditionally trained research funding reviewers’ point of view, research approaches will thus seem more familiar. The donor’s perceived risk associated with the project will appear smaller than a transdisciplinary one, thus increasing the likelihood for funding. The inclusion of stakeholders is central, both to increase the funding opportunity, and the relevance and effectiveness of transdisciplinary knowledge production. This adaptation strategy will also increase the number of scientific publications, which increases funding chances in the next iteration. Successful transdisciplinary projects had been able to develop both research agendas and projects that involved multiple disciplines, and different stakeholders while retaining sufficient traditional disciplinary legitimacy.

Third, complexity is an intrinsic feature of many pressing environmental problems. This feature requires stakeholder participation both in the framing of transdisciplinary research issues, and in the actual research (cf. Funtowics and Ravetz 1992). Thus stakeholder participation furthers solving complex problems and, in turn, reinforces the funding chances of transdisciplinary research. Note, however, that the number of transdisciplinary researchers may be a limiting factor to the growth of this field as desired in research policy.

Fourth, but operating at the level of a research group and individual researcher, effective transdisciplinary knowledge production is dependent on the development of a functional transdisciplinary research team (Fig. 2b). The actual composition of such a team is governed by the issues at hand, but will require both academic specialists and lay competence from various stakeholder groups and interests. It is noteworthy that transdisciplinary approaches and ad hoc team formation is only likely when traditional governance or traditional research has failed to deliver solutions to pressing complex problems. Effective team development is a challenge because researchers are generally trained in traditional disciplinary perspectives and methodologies, and stakeholders are usually not trained in and lack experience in research. Several factors influence the development of an effective functional transdisciplinary research team. The model of what factors affect the delivery of transdisciplinary research (Fig. 2a) is linked to the team development model (Fig. 2b) through “Effective TD knowledge production” in the former and “TDR (Transdisciplinary research”).

## Discussion

### Barriers and Bridges for Transdisciplinary Knowledge Production

While transdisciplinary research is considered an important aspect of SD towards sustainability, the concept is complex, and its application is still under debate and development (Hirsch Hadorn et al. 2008; Bergmann et al. 2013). Research on SD and sustainability in social–ecological systems focuses on links among sub-systems, and emphasizes reciprocal interactions and feedbacks. However, it also needs to meet the challenge of interactions both within-scale and cross-scale between social and ecological components. These links and loops can be positive or negative and can lead to acceleration or deceleration in rates of change of all components and their interactions (Alberti et al. 2003; Liu et al. 2007). To address and focus research attention on the dynamic links in coupled systems, novel research methods appear as necessary (Liu et al. 2010; Angelstam et al. 2013a).

However, transdisciplinary research involves a number of potential obstacles, many of which originate from the fact that people from different scientific disciplines and academic traditions need to collaborate, integrate their knowledge and learn together to create something additional to what they normally do (Hirsch Hadorn et al. 2008). An effective integration of various participants from society, often with conflicting interests, is another challenge, requiring cooperative development of frameworks, goals, and values (cf. Sverdrup et al. 2010). Transdisciplinary projects also face the problem that disciplinary evaluations of funding applications may neglect the true transdisciplinary aspects of such projects, leading to undervaluation (Bergmann et al. 2005; Leavy 2011). A key coping strategy to succeed with transdisciplinary research is to set one’s own problem-solving agenda as an evolving process, and then secure funding for specific projects that contribute to this agenda. Success is thus characterized by researchers being able to act as honest brokers among colleagues and stakeholders, and hence to practice collaborative leadership (Gray 2008). To conclude, our system analysis approach based on our joint pool of experiences, and a review of Hirsch Hadorn’s et al. (2008) propositions, indicate four groups of factors that promote the development of transdisciplinary research.

First, stakeholder participation in learning regarding both diagnosis and treatment of real-world problems is crucial. Stakeholders should represent different sectors, levels of governance, and a high level of stakeholder participation (Elbakidze et al. 2010; Sverdrup et al. 2010). This takes time (Axelsson et al. 2013b), and hence funding for transdisciplinary research need to have a longer duration than disciplinary research project. Given potential differences in stakeholder representation and empowerment, we believe that results from stakeholder group modeling should be viewed as hypotheses that need to be tested by independent empirical analyses. Second, to cope with the mismatch between research policy and funding practice, securing funding should be viewed as a process that co-ordinates and adapts a suite of disciplinary, development, and implementation projects to satisfy a transdisciplinary knowledge production agenda. Third, functional team development in transdisciplinary research is strengthened by experienced leadership, and multi-level collaboration as well as self-reflection and evaluation of the problem-solving process (Axelsson et al. 2011, 2013b). Fourth, to avoid formal and informal control by traditional disciplines, transdisciplinary research needs to be well understood among the participants. Due to these factors, transdisciplinary research faces difficulties in becoming established within existing university faculty and department structures. Therefore, we stress the need for both academic and non-academic members to establish a balance between periods of intense transdisciplinary collaboration with defined joint outputs, and periods with disciplinary and multi-disciplinary work (Hirsch Hadorn et al. 2008).

**Fig. 3:**
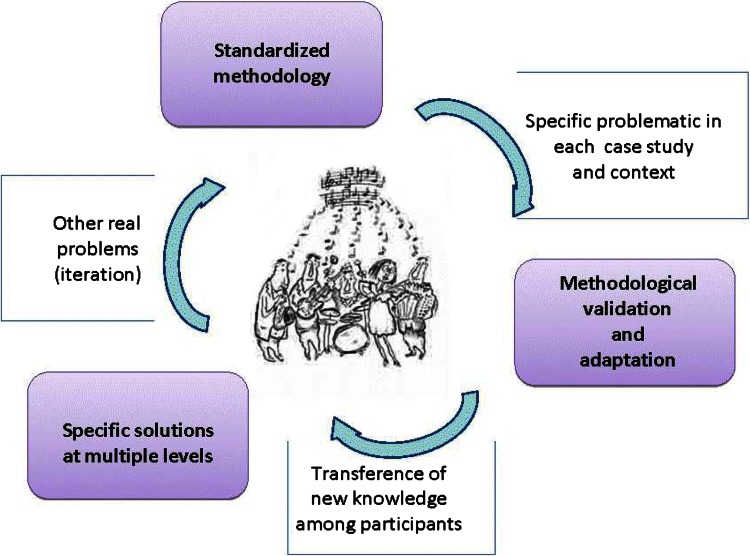
Cycle of re-enforcing transdisciplinary problem-solving capacity of a team of researchers, playing both individually and in concert like in jazz, from different disciplines and stakeholders, relevant to a particular issue

The problem-solving adaptive capacity of a team involved with transdisciplinary research increases with experience (Fig. 3). Thus by iteration, the methodology is improved towards enhanced transdisciplinary problem-solving capacity by adaptation and validation of the methodology in each new problem-solving case study. The development of a standardized methodology is particularly vital when aiming at meta-analyses of multiple case studies (Ostrom 2009; Hirsch Hadorn et al. 2008; Angelstam et al. 2013a).

### Green Infrastructures for Ecological Sustainability and Human Well-Being

Green infrastructure is a policy term that captures the need for functional ecosystems that deliver ecosystem services (European Commission 2010). Implementation of policies about green infrastructure includes many challenges to SD—a development that implies that finite resources and the environment are not consumed or degraded in an irrevocable manner, to the detriment of future generations. This implementation problem requires a transdisciplinary approach. To tackle the increasing loss and fragmentation of natural, semi-natural and cultural landscape land covers, and urban green space, there is a need to protect, manage, and restore functional habitat networks for wild life, ecosystem services, human health, and well-being (Grahn and Stigsdotter 2010; Sverdrup et al. 2010; Angelstam et al. 2011).

Simultaneously, however, production on forest and agricultural land is intensified, and more space is used for housing, industries and transport infrastructures in urban landscapes. As an example, the European forest-based sector has the vision by 2030 that it will be a key contributor to a sustainable European society (www.forestplatform.org). In a new, bio-based and customer-driven European economy, forestry is expected to make significant societal contributions. However, the national rural research strategy (FORMAS 2006) and the Swedish Government Rural Development Committee (Waldenström and Westholm 2009), have identified the potential increase in the demand for biological resources as a negative factor affecting rural Sweden’s ecological and social systems. Similarly, in urban landscapes green spaces shrink as roads and buildings expand (Tzoulas et al. 2007), which presents a threat to human health and well-being (Björk et al. 2008). Altogether, these trends imply increased conflicts between intensified economic use of forest and urban landscapes, and maintenance by protection, management, and restoration of functional green infrastructures for ecological sustainability. Similarly, as a stakeholder-driven modeling of environmental objectives and SD in the Swedish mountain areas indicated (Sverdrup et al. 2010), globalization and an increased demand on minerals, energy and other resources is likely to intensify future land-use conflicts and habitat fragmentation.

To achieve functional green infrastructures for ecological sustainability and human well-being, knowledge about species’ requirements, habitat and ecosystem processes are needed, as well as about effects on human health and well-being (Angelstam et al. 2004; Skärbäck 2007a, b; Annerstedt and Währborg 2011). Additionally, policies express different levels of ambition to be achieved in ecosystems (Angelstam et al. 2004; Svancara et al. 2005); e.g., (1) presence of species with small area requirements and generalists, (2) viable populations of species dependent on natural forest structures or having large area requirements, (3) ecological integrity with communities of all naturally occurring species and natural processes, and (4) social and ecological resilience. This includes aspects of promoting societal well-being and health as well as the core notion that sustainable natural resource management and governance are fundamental for public health in the surrounding community (Haines et al. 2009; Lederbogen et al. 2011; Sachs 2012). The human system therefore needs to be studied. Since neural pathways and synapses are changeable (e.g., brain plasticity) by for example environmental input, human beings are able to adapt their behavior to varied situations and experiences (Pascual-Leone et al. 2005). With functional magnetic resonance brain imaging it was found that urban upbringing as well as current urban living impact social evaluative stress processing in humans. The amygdala, a key region in the brain for regulation of negative affect and stress, was more active in the urban population compared to a rural one, making the urban people more vulnerable to stress (Lederbogen et al. 2011). This demonstrates distinct neural mechanisms for environmental risk factors. It is plausible that parallel mechanisms exist for the calming, stress-reducing effects of green environments.

Supporting implementation of green infrastructure policy requires informed collaborative and evidence-based spatial planning across sectors and levels of governance in forest, rural, and urban landscapes. Because panaceas generally do not work, comprehensive studies of complex, multivariable, non-linear, cross-scale, and changing social–ecological systems are needed case by case (Holling 1978). To contribute to functional green infrastructures and conflict resolution we argue for a dual approach. The first part concerns diagnosis in terms of how societal actors steer green infrastructures’ functionality by spatial planning, outputs related to planning processes and planning tools, as well as consequences on the ground for ecological sustainability and human well-being. This is consistent with the idea of applied institutional analysis or institutional diagnostics (Young 2013). The second part involves treatment in terms of production of socially robust knowledge about what functional green infrastructures require in terms of evidence-based knowledge about thresholds and tipping points in ecosystems (Rockström et al. 2009), and how to carry out governance, planning, and management of green infrastructures. This approach requires an understanding of global, social, and human system simultaneously (Opdam et al. 2006; sensu Komiyama et al. 2011).

To generate applicable knowledge about how to implement policies about green infrastructure, standardized studies of multiple social–ecological systems in different social–ecological contexts should be performed. This requires a multiple case study approach (Angelstam et al. 2013a) with comparative studies in key gradients representing global systems (e.g., different human footprints), social systems (e.g., institutions, power, and ownership patterns), and human systems (e.g., cultures). With its steep gradients in of all these dimensions, the European continent (Angelstam et al. 2013b) is particularly suitable. As already Marsh (1864) pointed out, understanding the role of history is crucial. This applies to the application of transdisciplinary research approaches both in regions that have not yet been severely impacted, and those that are severely impacted, and where rehabilitation, restoration, and re-creation are needed.

## Electronic supplementary material

Below is the link to the electronic supplementary material.

Supplementary material 1 (PDF 200 kb)
