# Supplementary material for: Solving Problems in Social–Ecological Systems: Definition, Practice and Barriers of Transdisciplinary Research
Source: Ambio. 2013 Mar 10;42(2):254–65. doi: 10.1007/s13280-012-0372-4 (PMC3593036; doi:10.1007/s13280-012-0372-4)
Supplement: Supplementary file 1 — Supplementary material 1 (PDF 200 kb) [file 13280_2012_372_MOESM1_ESM.pdf]

Electronic Supplementary Material

Solving problems in social-ecological systems:
definition, practice and barriers of transdisciplinary research

Per Angelstam, Kjell Andersson, Matilda Annerstedt, Robert Axelsson, Marine Elbakidze, Pablo Garrido, Patrik Grahn, K. Ingemar Jönsson, Simen Pedersen, Peter Schlyter, Erik Skärbäck, Mike Smith, Ingrid Stjernquist

Description of case studies

1. Trophic interactions among predators, prey and vegetation

A long history of boreal forest use and management in Sweden has promoted conifer-dominated forests at the expense of deciduous trees such as *Populus tremula*, *Salix caprea* and *Sorbus aucuparia*. As an abundant browser on young trees, the moose (*Alces alces*) is a key species both with respect to the maintenance of bird, insect, lichen, moss and species associated with these deciduous trees, and to the production of good quality Scots pine (*Pinus sylvestris*) timber (Angelstam et al. 2002). For the conservation of species there is a need to restore the deciduous forest component, which is also the preferred food of moose. If the ratio of moose density vs. amount of preferred food is too high, and hence browsing on the preferred tree species too intensive, this restoration can be difficult. To study the interactions between the abundance of preferred moose food, moose density and damage to trees, it is necessary to include landscapes with a broader combination of food abundance and moose density than found just in Sweden (Angelstam et al. 2000).

Angelstam, P. 2002. Large mammals, people, and the landscape - can trophic interactions be managed? In *Wildlife, land and people: priorities for the 21st century,* R. Field, R.L. Warren, H. Okarma, and P.R Sievert, 54-59. Bethesheda, MD: The Wildlife Society.

Angelstam, P., P.E Wikberg, P. Danilov, W.E. Faber, and K. Nygrén.2000. Effects of moose density on timber quality and biodiversity restoration in Sweden, Finland and Russian Karelia. *Alces* 36:133-145.

2. Brown bears and forest reindeer herding in Lapland

Due to conservation policies and implementation programs in combination with successful management strategies, large carnivore populations are gradually recovering in many parts of the world (Mech 1995; Linnell et al. 2001). These recently re-colonized areas encroach on an established human population, and consequently predator species can come into conflict with human interests (Ernest and Boyce 2000) and eventually affect local communities in areas where both humans and carnivores coexist (Cozza et al. 1996; Mishra 1997; Thirgood et al. 2005; Woodroffe et al. 2005). This situation is particularly critical in the Swedish reindeer husbandry region, especially in forested herding areas. Brown bear (*Ursus arctos*) predation on reindeer (*Rangifer tarandus*) calves is causing substantial impact on forest reindeer herds, forcing governmental authorities to find viable alternatives to contribute to functional conflict resolution. An alternative to increased governmental subsidies and compensation payments is requested from decision makers, carnivore managers and reindeer herders.

Cozza, K., R. Fico, M.L. Battistini, and E. Rogers. 1996. The Damage-conservation Interface Illustrated by Predation on Domestic Livestock in Central Italy. *Biological Conservation* 78: 329–336.

Ernest, H.B., and W.M. Boyce. 2000. DNA Identification of mountain lions involved in livestock predation and public safety incident. In *Proceedings of the 19th Vertebrate Pest Conference*, ed. T.P. Salmon, and A.C. Crab. University of California: Davis.

Mech, L.D. 1995. The challenge and opportunity of recovering wolf populations. *Conservation Biology* 9: 269-277.

Mishra, C. 1997. Livestock depredation by large carnivores in the Indian trans-Himalaya: conflict perceptions and conservation prospects. *Environmental Conservation* 24: 338-343.

Linnell, J.D.C., J.E. Swenson, and R. Andersen. 2001. Predators and people: conservation of large carnivores is possible at high human densities if management policy is favorable. *Animal Conservation* 4: 345-349.
Thirgood, S., R. Woodroffe and A. Rabinowitz. 2005. The impact of human-wildlife conflict on human lives and livelihoods. In *People and wildlife: conflict or coexistence?* ed. R. Woodroffe, S. Thirgood, and A. Rabinowitz, 13-26. London: Cambridge University Press.
Woodroffe, R., P. Lindsey, S. Romanach, A. Stein, and S. Ranah. 2005. Livestock predation by endangered African wild dogs (Lycaon pictus) in northern Kenya. *Biological Conservation* 124: 225-234.

3. Moose hunting and wolves in Norway

The main sources of conflict between hunters, wolves and moose hunting in Norway are that wolves kill many moose, that hunters are therefore forced to reduce their harvest, and that wolves kill hunting dogs (Skogen and Krange 2003; Storaas et al. 2008). The Koppangskjølen wolf pack established in Stor-Elvdal, Hedmark County in 1996/1997 and resided there for eight years. In 2001 the Directorate for Nature Management "leased" the hunting rights from the landowners for 0.5 million NOK for a 1-year pilot project. Although the lease was much lower than the expected economic losses, landowners felt their arguments were heard (Storaas et al. 2008). However, this compensation lasted one hunting season and the remaining wolves were shot through licensed hunting in 2005 (Storaas et al. 2008). Even though landowners do not have legal ownership of the moose, and although a controversial idea, the wolf conflict may be reduced if landowners are compensated for the economic loss of housing wolves (Storaas et al. 2008).

Skogen, K., and O. Krange. 2003. A wolf at the gate: The anti-carnivore alliance and the symbolic construction of community. *Sociologia Ruralis* 43:309-325.
Storaas, T., S. Pedersen, H.P. Andreassen, J.M. Arnemo, A. Eriksen, A. Frugaard, H. Gundersen, T. A. Haug, et al. 2008. Effects of wolves on moose: when the wolf came and went from Koppangkjølen. Elverum: Hedmark University College.

4. Protected area networks' functionality in Sweden

Swedish policies pronounce that naturally occurring species should be maintained in viable populations. This requires maintenance of functional networks of habitats. Angelstam et al. (2011) reviewed (1) the policy implementation process, (2) outputs in terms of the development of a hierarchical spatial planning approach, and (3) consequences for functionality of forest habitat networks and collaboration among stakeholders. The review showed that representation of different ecosystems is poor (Nilsson and Götmark 1992), the amount of different habitats protected is low (Angelstam and Andersson 2001), and that stakeholders have opposing opinions about the extent to which protected areas form green infrastructures (e.g., Eriksson and Hammer 2006). Focusing on three Swedish counties (Angelstam et al. 2003, Länsstyrelsen Östergötland 2007) as examples of close collaboration among academic and non-academic actors, and innovative combinations of funding sources, transdisciplinary practice facilitated implementation of policy strategies. However, systematic scaling up of knowledge-based collaboration that integrates protection, management and restoration is needed.

Angelstam, P., and L. Andersson. 2001. Estimates of the needs for forest reserves in Sweden. *Scandinavian Journal of Forest Research* Supplement No. 3: 38-51.

Angelstam, P., G. Mikusinski, J.A. Eriksson, P. Jaxgård, O. Kellner, A. Koffman, B. Ranneby, J-M. Roberge et al. 2003. *Gap analysis and planning of habitat networks for the maintenance of boreal forest biodiversity in Sweden - a technical report from the RESE case study in the counties Dalarna and Gävleborg*. Länsstyrelserna i Dalarna och Gävleborg, report 26 and 12. Falun and Gävle, Sweden.
Angelstam, P., K. Andersson, R. Axelsson, M. Elbakidze, B.-G. Jonsson, and J.-M. Roberge. 2011. Protecting forest areas for biodiversity in Sweden 1991-2010: policy implementation process and outcomes on the ground. *Silva Fennica* 45(5): 1111–1133.
Eriksson, S., and M. Hammer. 2006. The challenge of combining timber production and biodiversity conservation for long-term ecosystem functioning - A case study of Swedish boreal forestry. *Forest Ecology and Management* 237(1-3): 208-217.
Länsstyrelsen Östergötland 2007. *Levande eklandskap i Östergötland – regional landskapsstrategi 2008-2015 [Living oak landscapes in Östergörland – regional landscape landscape strategy 2008-15]*. Rapport 2007:22. Linköping: Länsstyrelsen Östergötland. (in Swedish).
Nilsson, C., and F. Götmark. 1992. Protected areas in Sweden: is natural variety adequately represented? *Conservation Biology* 6: 232–242.

5. Spatial planning for habitat networks in Scotland

The aim of the Central Scotland Green Network (CSGN) is to incorporate ecological networks (Bruinderink et al. 2003) into the planning system to deliver a strategic framework for functioning integrated habitat networks (IHN). The development of Forest Research's IHN model (Watts et al. 2010) in the CSGN was achieved with stakeholder involvement through a range of externally funded projects. The initial project was funded by the Glasgow and Clyde Valley Green Network Partnership in 2007. The enabling of those involved in the spatial planning process to take up the network approach was realized through participatory processes (Eycott et al. 2011). This entailed interactions with relevant actors, including discussions on the application of landscape ecological theory to address practical conservation issues. This ensures that local priorities are reflected in the IHN outputs and that actors involved feel joint ownership in the outputs, so that biodiversity planning has a more prominent role in the decision making process.

Bruinderink, G.G., T. Van Der Sluis, D. Lammertsma, P. Opdam, and R. Pouwels. 2003. Designing a coherent ecological network for large mammals in north western Europe. *Conservation Biology* 17(2): 549-557.
Watts, K., A.E. Eycott, P. Handley, D. Ray, J.W. Humphrey, and C.P. Quine. 2010. Targeting and evaluating biodiversity conservation action within fragmented landscapes: an approach based on generic focal species and least-cost networks. *Landscape Ecology* 25(9): 1305-1318.
Eycott, A. E., M. Marzano, and K. Watts. 2011. Filling evidence gaps with expert opinion: The use of Delphi analysis in least-cost modelling of functional connectivity. *Landscape and Urban Planning* 103(3-4): 400-409.

6. Swedish Environmental Objective "Magnificent Mountains"

The Swedish Environmental Objectives (EO) should guide activities among all economic sectors, stakeholders and the public at large (SOU 2000:52). Implementation is not only top-down, but dependent on aggregated action of many actors. Progress towards the objectives has varied. Criticism has been leveled against the EOs (Emmelin and Lerman 2004; RIR

2005) concerning both the balance in resource allocation between monitoring and reporting, and resources for actual environmental improvement. Limited progress with regard to the "Magnificent Mountain Landscapes" objective in central and northern Sweden led to a transdisciplinary study in order to better understand the underlying causes (Sverdrup et al. 2010; Schlyter et al. 2012; Stjernquist et al. 2012). Group modeling revealed the need for improvement of perceived legitimacy as well as for wider acceptance and support among stakeholders if the environmental objectives are to be attained.

Emmelin, L., and P. Lerman. 2004. *Environmental regulations – obstacles for development and a good environment?* Centre for Spatial Development and Planning Report 2004:09, 143 pp. Karlskrona: Blekinge Institute of Technology Research. (in Swedish).

Schlyter, P., I. Stjernquist, and H. Sverdrup. 2012. Handling Complex Environmental Issues – Formal Group Modelling as a Deliberative Platform at the Science-Policy-Democracy Interface. Proceedings of the 30th International Conference of the System Dynamics Society, July 22-26, 2012, St. Gallen, Switzerland. Available at http://www.systemdynamics.org/conferences/2012/proceed/papers/ P1405.pdf.

SOU. 2000:52. Framtidens miljö-allas vårt ansvar [The future environment – the responsibility of us all]. Betänkande från Miljömålskommittén. Stockholm: Miljödepartementet (in Swedish).

Stjernquist, I., H. Sverdrup, P. Schlyter, S. Belyazid, D. Koca, and U. Jönsson-Belyazid. 2012. Searching for the Magnificent Mountain Landscapes – On finding complex sustainability issues in a simple environmental problem. Proceedings of the 30th International Conference of the System Dynamics Society, July 22-26, 2012, St. Gallen, Switzerland. Available at http://www.systemdynamics.org/conferences/2012/proceed/papers/ P1408.pdf

Sverdrup, H., S. Belyazid, D. Koca, U. Jönsson-Belyazid, P. Schlyter, and I. Stjernquist. 2010. *Miljömål I fjällandskapet- en syntes av problemställningar knutna till förvaltningen av en begränsad resurs [Environmental Objectives in the Mountain Landscape. A synthesis of issues associated with the management of a limited resource].* Naturvårdsverket Rapport 6366, 165 pp. Stockholm: Naturvårdsverket (in Swedish).

RIR 2005. *Miljömålsrapporteringen - för mycket och för lite [The Environmental Objectives Reporting – too much and too little].* Stockholm: Riksrevisionen (in Swedish).

7. Cultural and natural values in road planning

The TransportMistra research program aimed to contribute to more sustainable transport systems. The INCLUDE sub-program focused on the inclusion of natural and cultural values in planning for transport infrastructure (e.g., Antonson et al. 2010; Mikusinski et al. 2012). After the first of two planned 3-year phases, the project was terminated due to poor scientific quality, poor collaboration with end-users and a failure to present plans for useful applications. In response to this, researchers initiated an evaluation led by an external researcher who interviewed researchers, representatives for end-users, and the donors. The result showed that the sub-program management, end-users and many researchers had a poor initial understanding of transdisciplinary research, scientific leadership failed in creating a creative space for researchers and end-users, and that integration between and within researchers and end-user groups was poor. To conclude, transdisciplinary knowledge production processes require much time, needs facilitation, and should initially not be too complex with too many actors and activity levels.

Antonson, H., M. Gustafsson, and P. Angelstam. 2010. Cultural heritage connectivity. A tool for EIA in transportation infrastructure planning. *Transportation Research Part D* 15: 463–472.
Mikusiński, G., M. Blicharska, H. Antonson, M. Henningsson, G. Göransson, P. Angelstam, A. Seiler. 2012. Integrating ecological, social and cultural dimensions in the implementation of the Landscape Convention. *Landscape Research*. doi: 10.1080/01426397.2011.650629

8. Geographic Information Systems to support decisions

Policies about forests and landscapes in the EU and Sweden stress the need for increased production of renewable raw materials (Regeringens Proposition 2007/08; Larsson et al. 2009), to support people's ability to pursue outdoor activities (Regeringens Proposition 2009/10), and to support functional green infrastructures for ecological and socio-cultural values (Carlgren and Löfroth 2010; European Commission 2010). The need to include both ecological and social systems in sustainable development processes has implications for spatial planning for all sectors (Opdam et al. 2006), and thus to integrate various specialized areas of expertise (Asplund and Hilding-Rydevik 2001). Geographic Information Systems (GIS) provides tools for data interpretation, landscape modeling, and visualizations that provide actors and stakeholders of different backgrounds in planning processes a common language for communication of indicators, targets, and impacts (Rambaldi et al. 2006; Zetterberg 2009). However, to realize the potential of GIS to produce new information that can be used for collaborative learning processes, better integration of data, algorithms and communication is needed (Andersson 2011).

Andersson, K. 2011. Geographic information systems as a tool to support monitoring and assessment of landscape and regional sustainability. Acta Universitatis Agriculturae Sueciae 2011:92. Uppsala: Swedish University of Agricultural Sciences.
Asplund, E., and T. Hilding-Rydevik. 2001. Arena för hållbar utveckling, aktörer och processer. [Arena for sustainable development, actors and processes]. Stockholm: KTH Royal Institute of Technology. (in Swedish).
Carlgren, A., and M. Löfroth. 2010. Uppdrag om förstudie om uppbyggandet av grön infrastruktur och framtagande av indikatorer för gynnsam bevarandestatus. [Plan for feasibility study about building green infrastructure and development of indicators for favorable conservation status.] Stockholm: Regeringsbeslut M2010/3407Na (in Swedish).
European Commission. 2010. Green Infrastructure Implementation. Proceedings of the European Commission Conference 19 November 2010, Brussels, Belgium.
Larsson, S., T. Lundmark, and G. Ståhl. 2009. Möjligheter till intensivodling av skog. [Opportunities for intensive production of forest]. Slutrapport från regeringsuppdrag Jo 2008/1885, Uppsala, Sweden (in Swedish).
Rambaldi, G., P.A. Kwaku Kyem, M. McCall, and D. Weiner. 2006. Participatory Spatial Information Management and Communication in Developing Counties. *The Electronic Journal of Information Systems in Developing Countries* 25(1): 1-9.
Regeringens Proposition 2007/08:108. En skogspolitik i takt med tiden. [A contemporary forest policy.] Regeringens proposition, Stockholm, Sweden (in Swedish).
Regeringens Proposition 2009/10:238. Framtidens friluftsliv. [Future recreation], Regeringens proposition, Stockholm, Sweden (in Swedish).
Zetterberg, A. 2009. Network based tools and indicators for landscape ecological assessments, planning, and design. Licentiate thesis. Stockholm: KTH Royal Institute of Technology.

9. Land consolidation in Dalarna County, Sweden

In the past, when land was inherited it was divided among heirs (Sporrong 1994). As a consequence, land became fragmented but gave the region a distinct culture, social capital, level of entrepreneurship and an attractive cultural landscape (Westholm 1992). When forestry and agriculture became industrialized (Antonson and Jansson 2011) land fragmentation was seen as a problem. Efforts aiming to deal with this in Dalarna county lagged behind those in other parts of Sweden, but have recently been intensified (Länsstyrelsen Dalarna 2010). The land consolidation has been a top-down process, aiming to convince private land owners about land consolidation. The states' will to pursue the land consolidation process involves "forest political, regional and other society-economical motives" (Anon. 2003). However, there is an ongoing transition to use land consolidation as a social learning process that considers all sustainability dimensions.

Anon. 2003. Samlad skog [Forest in one place]. Länsstyrelsen Dalarna, 6 pp. Lantmäteriet och Skogsvårdsstyrelsen Dalarna-Gävleborg.
Antonson, H., and U. Jansson. 2011. *Agriculture and forestry in Sweden since 1900. Geographical and historical studies*. Stockholm: Royal Swedish Academy of Forestry and Agriculture.
Länsstyrelsen i Dalarna. 2010. Långsiktig plan för omarrondering i Dalarnas län [Long-term plan for land consolidation in Dalarna County]. Rapport 2010:18, 12 pp. Falun, Sweden.
Sporrong, U. 1994. Agrarian landscapes in Sweden that are of particular scientific interest. *Bebyggelsehistorisk tidskrift* 26: 71-90
Westholm, E. 1992. Mark, människor och moderna skiftesreformer i Dalarna. [Land, people and modern land consolidation reforms in Dalarna] Geografiska Regionstudier 25. Uppsala: Uppsala Universitet.

10. Creation of the Roztochya Biosphere Reserve in Ukraine

The Biosphere Reserve (BR) concept seeks to simultaneously reconcile and promote conservation of natural and cultural diversity, environmentally and socio-culturally sustainable economic development, and research (UNESCO 1995; Elbakidze et al. 2013). The Roztochya BR is located in Western Ukraine. It was established after many years of intensive discussion and of numerous attempts of BR-promoters to garner the acceptance of local communities. The study of Roztochya BR development and establishment (Elbakidze et al. 2012) shows that implementation of the BR as "learning sites for sustainable development" (UNESCO 2008) benefits if: (1) the national terminology describing BRs is chosen carefully because it affects how it is perceived by stakeholders; (2) the legislative interpretation of BR in national legislation reflects its multi-sectoral character; (3) stakeholders who implement local BR initiatives have the understanding, knowledge and will to lead and facilitate SD as a collaborative social learning process towards ecological, economic, social and cultural sustainability.

Elbakidze, M., P. Angelstam, C. Sandström, N. Stryamets, S. Crow, R. Axelsson, G. Stryamets, and T. Yamelynets. 2013. Biosphere reserve for conservation and development in Ukraine? Legal recognition and a case study of establishment. *Environmental Conservation*. doi: 10.1017/S0376892912000434

Elbakidze, M., T. Hahn, V. Mauerhofer, P. Angelstam, R. Axelsson. 2013c. Legal framework for biosphere reserves as learning sites for sustainable development: a comparative analysis of Ukraine and Sweden. *AMBIO* 42(2): 174–187.
UNESCO. 1995. The Seville Strategy and the Statutory Framework of the World Network of Biosphere Reserves. Paris: UNESCO.
UNESCO. 2008. Madrid Action Plan for Biosphere Reserves (2008-2013). Paris: UNESCO.

11. Public procurement of food with an environmental profile

Public procurement has been proposed as a driver for environmental improvement as well as product and technological development (Pedersen 2011; SOU 2011). Food production has major direct and indirect environmental as well as health impacts. Improved environmental performance through procurement is of clear societal interest. Nevertheless, when procuring organizations' attempt to stipulate environmental requirements, they tend be challenged in court to an unprecedented degree. The Swedish Environmental Procurement Board, which is tasked with developing and supporting environmental public procurement, could not understand why the food and meals sector was so conflict ridden compared to other procurement sectors. Hence it commissioned a transdisciplinary study to better understand the difficulties (Schlyter et al. 2011). The main driver of litigation was, unexpectedly to stakeholders, to be found in the dynamics of the public procurement, i.e. the system dynamics as such, rather than the will of any individual actor.

Pedersen, K. 2011. Upphandlingens grunder – en introduktion till offentlig upphandling och upphandlingen i försörjningssektorerna. [The basics of procurement - an introduction to public procurement and the procurement in supply sectors]. 2a upplagan. Stockholm: Jure Förlag AB (in Swedish).
Schlyter, P., I. Stjernquist, H. Sverdrup, and S.C.W. Johansson. 2011. A group modelling based analysis of public procurement of food and meals: obstacles, problems and opportunities. Miljöstyrningsrådet Rapport 2011:08, 50 pp. Stockholm: Miljöstyrningsrådet (in Swedish).
SOU 2011: 73. På jakt efter den goda affären – analys och erfarenheter av den offentliga upphandlingen. [Hunting for the good deal – analysis and experiences of public procurement]. Stockholm: Delbetänkande av upphandlingsutredningen 2010 (in Swedish).

12. Landscape character vs. health and wellbeing

Green environments benefits human health (Björk et al. 2008; Annerstedt 2011). Based on environmental psychology research (Grahn and Stigsdotter 2010) five environmental characteristics (serene, wild, lush, space and culture) were mapped with GIS (Skärbäck et al. 2012). Health and well-being is monitored regularly in Scania in south Sweden. Using the survey from year 2004 (n=25 000), Björk et al. (2008) reported associations between the number of characteristics and the level of neighborhood satisfaction. The survey in 2008 included also questions on how respondents perceived the five characteristics. An analysis by de Jong et al. (2012) found clear evidence that physical activity, general health and neighborhood satisfaction were associated with the perceived qualities culture, serene and lush. A study of the city Malmö shows that qualities within 300 m from respondents' residences were associated with their self-reported perception of health and well-being. Park size and indicators of green well-being qualities were correlated (Stoltz et al. unpubl.).

Annerstedt, M. 2011. Nature and public health - Aspects of promotion, prevention, and intervention. Doctoral Thesis. Alnarp: Swedish University of Agricultural Sciences.
Björk, J., M. Albin, P. Grahn, H. Jacobsson, J. Ardö, J. Wadbro, P.O. Östergren, and E. Skärbäck. 2008. Recreational values of the natural environment in relation to neighbourhood satisfaction, physical activity, obesity and wellbeing. *Journal of Epidemiology and Community Health* 62: e2 doi:10.1136/jech.2007.062414
de Jong, K., M. Albin, E. Skärbäck, P. Grahn, and J. Björk. 2012. Perceived green qualities were associated with neighborhood satisfaction, physical activity, and general health: results from a cross-sectional study in Scania, southern Sweden. *Health & Place* 18(6):1374-80
Grahn, P., and U.K. Stigsdotter. 2010. The relation between perceived sensory dimensions of urban green space and stress restoration. *Landscape and Urban Planning* 94: 264-275.

13. Stress, neurobiology, and green space management

Urbanization and disconnection with nature have disrupted the balance between human's biological conditions and how we behave. One consequence is maladaptive stress reactions, manifested as varied mental disorders (McEwen and Wingfield 2003, Lederbogen et al. 2011). In spite of many empirical studies showing associations between nature and stress relief (Bowler et al. 2010), neither health policies nor the medical agenda have been much influenced. One reason may be the lack of knowledge on the physiological mechanisms, and a more profound knowledge of complex gene-environment interactions. A recent transdisciplinary project involving medicine, psychophysiology, and virtual technology demonstrated a measurable positive effect on the parasympathetic nerve system by exposure to virtual nature and nature sounds. This is among the first studies demonstrating a possibility to record advanced physiological measures in a virtual nature setting, something that may inspire future studies on the interactive networks of risk factors involved in the ontogeny of disordered development and health. This would serve as a helpful contribution to epidemiological hierarchical regression models in detecting multiple interacting causal effects. Such approaches to complex systems of health and environment could potentially steer policies concerning matters from public health to natural resource management.

Bowler, D., L. Buyung-Ali, T. Knight, and A. Pullin. 2010. A systematic review of evidence for the added benefits to health of exposure to natural environments. *BMC Public Health* 10: 456.
Lederbogen, F., P. Kirsch, L. Haddad, F. Streit, H. Tost, P. Schuch, S. Wüst, J.C. Pruessner, et al. 2011. City living and urban upbringing affect neural social stress processing in humans. *Nature* 474: 498-501.
McEwen, B.S., and J.C. Wingfield. 2003. The concept of allostasis in biology and biomedicine. *Hormones and behavior* 43: 2-15.

14. Establishment of a rehabilitation garden

The global burden of neuropsychiatric diseases is a major threat to public health for the foreseeable future (Murray and Lopez 1996). In the EU, less than one third of all cases receive any treatment or rehabilitation, suggesting a considerable level of unmet needs (Wittchen et al. 2011). The growing number of sick leaves attributed to stress and work related factors require increased demands on effective rehabilitation programs. However, effects of traditional medical rehabilitation considering sickness absenteeism and work ability for this group are weak (Kuoppala and Lamminpää 2008). By contrast, nature assisted

rehabilitation shows promising results (Nilsson et al. 2011). Stakeholders in the Scania region in Sweden (Swedish Social Insurance Agency, Scania Regional Council, Swedish Public Employment Service) commenced a partnership with researchers in 2008. As a result a rehabilitation garden, including procedures and methods was developed, the impact of rehabilitation was studied, and a certification procedure established (Grahn et al. 2010).

Grahn, P., C. Tenngart Ivarsson, U.K. Stigsdotter, and I.-L. Bengtsson. 2010. Using affordances as a health-promoting tool in a therapeutic garden. In *Innovative approaches to researching landscape and health*, ed. C. Ward Thompson, S. Bell, and P. Aspinall, 116-154. London: Routledge.

Nilsson, K., M. Sangster, C. Gallis, T. Hartig, S. De Vries, K. Seeland, and J. Schipperijn. 2011. *Forests, trees, and human health*. Dordrecht: Springer Science.

Kuoppala, J., and A. Lamminpää. 2008. Rehabilitation and work ability: A systematic literature review. *Journal of Rehabilitation Medicine* 40: 796-804.

Murray, C.J.L., and A.D. Lopez. 1996. *Global Burden of Disease. A comprehensive assessment of mortality and disability from diseases, injuries, and risk factors in 1990 and projected to 2020*. Cambridge: Harvard School of Public Health.

Wittchen, H.U., F. Jacobi, J. Rehm, A. Gustavsson, M. Svensson, B. Jönsson, J. Olesen, C. Allgulander, et al. 2011. The size and burden of mental disorders and other disorders of the brain in Europe 2010. *European Neuropsychopharmacology* 21(9): 655-679.
